# Identification of senescence-related genes in diagnosing idiopathic pulmonary fibrosis via integrating bioinformatics analysis and machine learning

**DOI:** 10.1371/journal.pone.0353694

**Published:** 2026-08-07

**Authors:** Chenchun Ding, Ziwei Ye, Pu Zeng, Weibin Chen

**Affiliations:** 1 Department of Neurosurgery, Beijing Tsinghua Changgung Hospital, School of Clinical Medicine, Tsinghua Medicine, Tsinghua University, Beijing, China; 2 School of Pharmaceutical Sciences, Xiamen University, Xiamen, Fujian, China; 3 Department of Respiratory and Critical Care Medicine, Beijing Tsinghua Changgung Hospital, School of Clinical Medicine, Tsinghua Medicine, Tsinghua University, Beijing, China; 4 Department of General Surgery, Zhongshan Hospital of Xiamen University, School of Medicine, Xiamen University, Xiamen, Fujian, China; Texas A&M University College Station: Texas A&M University, UNITED STATES OF AMERICA

## Abstract

Idiopathic pulmonary fibrosis (IPF) is a chronic, progressive lung disease characterized by persistent alveolar epithelial injury and aberrant tissue remodeling. Increasing evidence suggests that senescence of alveolar epithelial cells (AECs) contributes to impaired epithelial regeneration and maladaptive tissue repair by limiting reparative capacity and promoting profibrotic signaling. However, the molecular drivers of AEC senescence and their impact on the immune microenvironment in IPF remain incompletely understood. Here, we investigated senescence-associated genes involved in IPF pathogenesis and evaluated their diagnostic and therapeutic potential. IPF transcriptomic datasets were retrieved from the Gene Expression Omnibus (GEO). Senescence-related differentially expressed genes (SRDEGs) were identified by intersecting IPF-derived differentially expressed genes with a curated human senescence gene list. Functional enrichment analyses were performed to delineate SRDEG-associated biological processes. Hub genes were prioritized using machine-learning approaches, and a diagnostic model was constructed and assessed by receiver operating characteristic (ROC) analysis. Candidate genes were further validated through in vivo and in vitro experiments. Given the upstream regulatory role of CHEK2 in DNA damage response-associated cellular senescence, Fostamatinib was screened as a potential therapeutic agent, and its interaction with CHEK2 and functional effects were examined using molecular docking, molecular dynamics simulations, and experimental assays. Two senescence-associated hub genes, CHEK2 and TP53 BP1, were identified as key contributors to IPF pathology (FDR-adjusted P < 0.05), and a model incorporating both genes achieved high diagnostic performance. Experimental validation, however, indicated that only CHEK2 showed IPF-specific differential expression and was closely associated with AEC senescence and fibrotic progression. In silico analyses supported stable binding between Fostamatinib and CHEK2, and subsequent molecular and cellular experiments suggested that Fostamatinib may attenuate CHEK2-associated senescence and profibrotic responses. Collectively, these findings identify CHEK2 as a critical regulator of AEC senescence and IPF development, supporting its potential use as a diagnostic biomarker and therapeutic target. Fostamatinib may represent a candidate therapeutic strategy for IPF by modulating CHEK2-related senescence pathways.

## 1. Introduction

Idiopathic pulmonary fibrosis (IPF) is an age-associated, irreversible interstitial lung disease (ILDs) with markedly elevated risk in elderly individuals and in patients carrying syndromic mutations that promote cellular senescence, such as telomerase component mutations [1]. Current epidemiological data estimate a global incidence of approximately 1–13 per 100,000 individuals and a prevalence of 3–45 per 100,000 [2,3], accounting for roughly one-third of all ILDs [4]. Among patients with end-stage disease, the median survival is 5.2–6.7 years after lung transplantation, whereas the median survival of advanced IPF patients without transplantation is less than 2 years [5]. High-resolution computed tomography (HRCT) remains the primary diagnostic modality; however, due to the complex pathogenesis of IPF, some patients with progressive disease lack typical radiological features [6]. Moreover, the current ATS/ERS/JRS/ALAT clinical practice guidelines have not yet incorporated blood-based biomarkers into diagnostic or therapeutic decision-making [7]. Therefore, the identification of effective biomarkers is of critical importance for the early diagnosis and treatment of IPF.

Alveolar epithelial cells (AECs) senescence and impaired regeneration are considered central to the onset and progression of IPF [8]. Senescent cells lose their proliferative capacity and secrete a wide array of pro-inflammatory cytokines (e.g., IL-1β, TNF-α), chemokines (e.g., CCL2, CCL3), and matrix metalloproteinases (e.g., MMP2, MMP28), collectively termed the senescence-associated secretory phenotypes (SASPs) [9,10]. These factors exacerbate injury to surrounding normal cells and induce epithelial–mesenchymal transition (EMT) [11–13], thereby accelerating the fibrotic process. Anti-fibrotic interventions targeting cellular senescence have shown promising outcomes in animal studies. For instance, senolytic agents such as quercetin and dasatinib, as well as SASPs inhibitors, can alleviate IPF [14–16]. However, the clinical translation of senescence-targeted findings into diagnostic and therapeutic applications for IPF remains limited, underscoring the need for deeper investigation of its molecular mechanisms and potential interventions from a senescence perspective.

Bioinformatics and machine learning have emerged as powerful tools for elucidating disease mechanisms and identifying key genes, showing considerable promise in biomarker discovery, cancer diagnostics, and prognostic evaluation [17–19]. To identify robust diagnostic biomarkers for IPF, this study integrates differential gene expression analysis, machine learning algorithms, molecular docking, and molecular dynamics (MD) simulations to screen senescence-related differentially expressed genes (SRDEGs) and potential therapeutic agents relevant to IPF progression. These findings aim to provide novel insights into the diagnosis and targeted treatment of IPF by focusing on senescence-associated molecular mechanisms.

## 2. Materials and methods

### 2.1. Data acquisition, processing, and differential expression analysis

Senescence-related genes were obtained from the Human Aging Genomic Resources (HAGR) (https://genomics.senescence.info/genes/index.html), including a total of 307 Senescence-related genes. IPF-related datasets were downloaded from the Gene Expression Omnibus (GEO) database (https://www.ncbi.nlm.nih.gov/geo/). Specifically, GSE10667 included 31 IPF samples and 15 normal samples(https://www.ncbi.nlm.nih.gov/geo/query/acc.cgi?acc=GSE10667) [20], GSE21369 contained 11 IPF samples and 6 normal samples (https://www.ncbi.nlm.nih.gov/geo/query/acc.cgi?acc=GSE21369) [21], GSE53845 had 40 IPF samples and 8 normal samples (https://www.ncbi.nlm.nih.gov/geo/query/acc.cgi?acc=GSE53845) [22], and GSE110147 comprised 22 IPF samples and 11 normal samples (https://www.ncbi.nlm.nih.gov/geo/query/acc.cgi?acc=GSE110147) [23]. GSE24206 was used as a validation dataset, including 6 normal and 17 IPF tissue samples (https://www.ncbi.nlm.nih.gov/geo/query/acc.cgi?acc=GSE24206) [24]. Data normalization and differentially expressed genes (DEGs) analysis were performed using the “limma” package in R version 4.4.1. Volcano plots were generated using the “ggplot2” package.

### 2.2. Differential expression analysis of age-related genes

SRDEGs between DEGs and senescence-related genes were identified using the “venn” and “limma” packages. SRDEGs were defined by an adjusted p-value < 0.05 and |log2-fold-change| > 0.5. The adjusted p-value threshold was used to control the false discovery rate, whereas the |log2-fold-change| > 0.5 cutoff was selected to retain moderate but potentially biologically relevant changes within the restricted senescence-related gene set while excluding minimal expression fluctuations. Heatmaps were generated using the “heatmap” function in R.

### 2.3. Functional enrichment and protein-protein interaction network analysis of SRDEGs

Disease ontology (DO), gene ontology (GO) and Kyoto Encyclopedia of genes and genomes (KEGG) analyses of SRDEGs were performed using the “ClusterProfiler” R package. The STRING database (https://string-db.org/) was used to visualize the PPI network among SRDEGs.

### 2.4. Identification of potential biomarkers in normal and IPF samples

Biomarkers for IPF were identified using machine learning algorithms: least absolute shrinkage and selection operator (LASSO), support vector machine–recursive feature elimination (SVM-RFE), and Random Forest (RF). The “glmnet” package in R was used for LASSO, the “e1071” package for SVM-RFE, and the “randomForest” package for RF. Overlapping genes identified by these algorithms were considered as hub age-related genes for IPF diagnosis.

### 2.5. Identification and validation of hub age-related genes

The expression levels of hub genes were evaluated using box plots to compare normal and IPF individuals. Receiver operating characteristic (ROC) curves were plotted using the “pROC” package in R to assess the diagnostic accuracy of hub age-related genes in both training and test datasets, with GSE24206 serving as an external validation dataset.

### 2.6. Molecular docking

Drug-gene interactions were explored using DrugBank (https://go.drugbank.com/) to identify drugs associated with key genes. To assess whether Fostamatinib could interact with the relevant target involved in AEC senescence, molecular docking was performed between Fostamatinib and CHEK2. First, the three-dimensional (3D) structure of the human target protein was obtained from the Protein Data Bank (PDB, https://www.rcsb.org/), and the two-dimensional (2D) structure of Fostamatinib was retrieved from the PubChem database (https://pubchem.ncbi.nlm.nih.gov/). Subsequently, binding energy (kcal/mol) was calculated following the standard docking procedure of AutoDock v1.2.3. The docking grid box was set to 50 Å in the X, Y, and Z directions to encompass the kinase-domain binding pocket and adjacent residues, thereby allowing ligand conformational flexibility while avoiding unrestricted whole-protein docking. The genetic algorithm was executed 10 times. The ligand–receptor interactions were then analyzed in detail using PyMOL v2.6 and LigPlot+ v2.2.8 to generate 3D interaction diagrams. The 2D interaction patterns of the protein–ligand complex were visualized using Leview software.

### 2.7. Molecular dynamics

MD simulations were performed using GROMACS 2022.3 [25]. Small-molecule preprocessing was conducted with AmberTools 22, in which the GAFF force field was applied. Hydrogen atoms were added and the RESP electrostatic potential was calculated using Gaussian 16W, and the resulting electrostatic potential data were incorporated into the topology file of the MD system. The simulation system was constructed under conditions of 300 K constant temperature and 1 bar constant pressure, employing the Amber99sb-ildn force field with the TIP3P water model. To ensure overall charge neutrality, an appropriate number of Na⁺ ions were added. The system was first subjected to energy minimization using the steepest descent algorithm, followed by NVT (constant number of particles, volume, and temperature) and NPT (constant number of particles, pressure, and temperature) equilibration for 100,000 steps each, with a coupling constant of 0.1 ps and a total equilibration time of 100 ps. After the system reached equilibrium, unrestrained MD simulations were performed for a total of 5,000,000 steps with a 2 fs time step, corresponding to an overall simulation time of 100 ns. Post-simulation trajectory analyses were conducted using the built-in GROMACS analysis tools to calculate the root-mean-square deviation (RMSD), root-mean-square fluctuation (RMSF), solvent-accessible surface area (SASA) and radius of gyration (Rg) of the protein and its amino acid residues. In addition, MM/GBSA free energy calculations and free energy landscape (FEL) analyses were performed to comprehensively evaluate the binding free energy and conformational stability of the protein–ligand complex.

### 2.8. Cell culture

A549 cells were purchased from ATCC and cultured in A549-specific medium (CM-0016, Pricella). Cells were maintained at 37°C in a 5% CO2 atmosphere (WH-15, WIGGENS). Cells were seeded at a density of 3 × 10^4^ cells per well and treated with 25μM BLM (11-B608166, Boer).

### 2.9. Quantitative real-time PCR analysis

Total RNA was extracted from A549 cells using TRIzol reagent (Catalog No. YZ-15596018, Acmec). The concentration of RNA was determined using a NanoDrop One ultramicro spectrophotometer (Thermo Fisher Scientific). cDNA was synthesized from the extracted RNA using the Hifair® II Reverse Transcriptase (Catalog No. 11110ES92*, Yeasen). Quantitative real-time PCR analysis (qRT-PCR) was conducted utilizing the Hieff® qPCR SYBR Green Master Mix (Catalog No. 11203ES08, Yeasen). GAPDH served as the normalizer. The primers for qRT-PCR were obtained from Baijin Biotechnology. The sequences of the primers used are as follows: human GAPDH: forward: 5′-GGAGCGAGATCCCTCCAAAAT-3′, reverse: 5′-GGCTGTTGTCATACTTCTCATGG-3′; human CHEK2: forward: 5′-GAGAGTGTGCGGCTCCAG-3′, reverse: 5′-GACTGTGAGGAGGAGCCTTG-3′; human TP53BP1: forward: 5′-CACAGAAAGTCCTCGTGCCT-3′, reverse: 5′-CTCTCCTGCCCCTACAGGTT-3′. All results are presented as n = 3, representing three biological replicates from independent samples.

### 2.10. Western blot analysis

Total protein was extracted using RIPA lysis buffer containing protease and phosphatase inhibitors. Proteins were separated by SDS-PAGE and transferred to PVDF membranes. Membranes were blocked with 5% skim milk, incubated with primary antibodies overnight at 4°C, washed, and then incubated with secondary antibodies. Band intensities were detected using a chemiluminescence imager (Bio-Rad). Primary antibodies used included CHEK2 (1:2000, ab175188, Abcam), TP53BP1 (1:2000, ab175933, Abcam), p16 (1: 2000, ab151303, Abcam), p21 (1: 2000, ab107099, Abcam), Sftpc (1: 2000, ab270521, Abcam), Fibronectin (Fn) (1: 2000, ab2413, Abcam), Collagen1a1 (Col1a1) (1: 2000, ab34710, Abcam), α-SMA (1: 2000, ab7817, Abcam), E-cadherin (1: 2000, 20874-1-AP, Proteintech), GAPDH (1: 2000, ab34710, Abcam), p-p53 (1: 2000, 28961-1-AP, Proteintech). Western blot results were quantified using ImageJ. After background subtraction, target protein signals were normalized to GAPDH and expressed as fold changes relative to the control group. Experiments were independently repeated at least three times. Data are shown as the mean ± SEM, and two-group comparisons were performed using an unpaired two-tailed Student’s t-test. P < 0.05 was considered statistically significant.

### 2.11. Animals

Male C57BL/6 mice (6 weeks old) were obtained from the Experimental Animal Center of Xiamen University. After acclimatization, mice were randomly assigned to the control, BLM, BLM+ Fostamatinib, and BLM+NIN groups using a random-number table (n = 6 mice per group). Pulmonary fibrosis was induced by intratracheal instillation of BLM (2 mg/kg; 11-B608166, Boer) dissolved in 40 μl sterile saline following anesthesia with 1% pentobarbital sodium (60 mg/kg). Control animals received an equal volume of sterile saline. Beginning on day 10 after BLM administration, mice were treated once daily with Fostamatinib (10 mg/kg; HY-13038A, MCE) dissolved in sterile saline and delivered by oral gavage. Nintedanib (NIN; 100 mg/kg, HY-50904, MCE) was used as the positive-control anti-fibrotic drug. Investigators performing histological staining, image acquisition, and quantitative tissue analysis were blinded to treatment allocation. Animal health and behavior were monitored twice daily, once in the morning and once in the evening. On day 20 after BLM exposure, mice were euthanized by gradual CO2 inhalation followed by cervical dislocation to ensure death before lung harvest. All experimental procedures were reviewed and approved by the Institutional Animal Care and Use Committee of Xiamen University (IACUC approval number: XMULAC20240170) and were conducted in accordance with the ARRIVE 2.0 guidelines and all relevant institutional and national regulations. All operators had undergone relevant training in animal experimental procedures.

### 2.12. Immunohistochemistry

Paraffin-embedded mouse lung tissues were sectioned into 5-µm slices using a microtome and subjected to immunohistochemistr (IHC) analysis. After deparaffinization and rehydration, sections were incubated overnight at 4 °C with primary antibodies against CHEK2 (1:60, ab175188, Abcam) and TP53BP1 (1:60, ab175933, Abcam). IHC staining was performed with a commercial detection kit (KIT-9720, MXB, China) according to the manufacturer’s instructions. Diaminobenzidine (DAB; Servicebio, China) was used as the chromogenic substrate. Slides were subsequently counterstained, mounted, and examined under a light microscope (Nikon SMZ1000).

### 2.13. Plasmid and cell transfection

The CHEK2 and control siRNAs were purchased from GenePharma (Suzhou, China). The target sequence for CHEK2 is 5′-TGTGTGAATGACAACTACT-3′.

### 2.14. Senescence-associated β-galactosidase staining

Cells were exposed to BLM for 72 h prior to staining. SA-β-gal activity was detected using a commercial SA-β-gal staining kit (G1580-100T, Solarbio) following the manufacturer’s protocol. The working solution was freshly prepared according to the kit instructions, and 6-well plates were sealed with plastic wrap to prevent evaporation during incubation. Cells were then incubated at 37 °C overnight, and senescent cells were identified as blue-stained cells under an optical microscope. For quantification, at least 5 randomly selected fields per well were counted by an investigator blinded to group allocation, and the percentage of SA-β-gal-positive cells was calculated as the number of blue-stained cells divided by the total number of cells × 100%.

### 2.15. Immunofluorescence staining

Cell immunofluorescence: A549 cells were cultured on glass coverslips and treated with 25 μM BLM for 72 h. After washing, cells were fixed in 4% paraformaldehyde, permeabilized with Triton X-100, and blocked with goat serum. Cells were then incubated overnight at 4 °C with the appropriate primary antibodies. Following additional washes, fluorescent secondary antibodies (Alexa Fluor® 594 or 488, 1:400; Invitrogen) and DAPI were applied. Coverslips were mounted with anti-fade medium and imaged using a high-sensitivity confocal laser scanning microscope (Zeiss LSM980).

Tissue immunofluorescence: Fresh lung tissues were cryosectioned and fixed in acetone, followed by blocking with goat serum for 20 min. Sections were incubated with properly diluted primary antibodies (1:60) overnight at 4 °C in a humidified chamber, then equilibrated at room temperature for 45 min. After washing, sections were incubated with Alexa Fluor® 594 or 488 secondary antibodies (1:400; Invitrogen) for 1 h and counterstained with DAPI. Slides were sealed with anti-fade mounting medium and visualized using a Zeiss LSM980 confocal microscope.

The primary antibodies included Ki67, E-cadherin, α-SMA, Colla1, Fn, and Sftpc (all from Abcam).

### 2.16. Cell viability assay

The effect of Fostamatinib on A549 cell proliferation was evaluated using the Cell Counting Kit-8 (CCK-8; AKCE001-1, BOXBIO). A549 cells were seeded in 96-well plates at a density of 5 × 10^3^ cells per well and allowed to adhere for 24 h. Cells were then treated with Fostamatinib at varying concentrations (2.5, 5.0, 7.5, and 10.0 μM) for 24 h. At the indicated time points, 10 μL of CCK-8 solution was added to each well and incubated at 37 °C for 2 h. The absorbance was measured at 450 nm using a microplate reader to determine cell viability.

### 2.17. Colony formation assay

After being seeded in 6-well plates at a density of 2 × 10^3^ cells per well and pretreated with 7.5 μM Fostamatinib for 24 hours, the cells were treated with 25 μM BLM for 72 hours. The cells were then fixed with 1 mL of 4% paraformaldehyde for 60 minutes, followed by washing and staining with 1 mL of crystal violet for 20 minutes prior to observation and photography.

### 2.18. ROS analysis

A549 cells were seeded in a 24-well culture dish and treated with BLM and Fostamatinib. DCFH-DA (50101ES01, Yeasen) working solution was prepared by diluting DCFH-DA in serum-free culture medium at a 1:1000 dilution. Cells were incubated at 37°C for 30 minutes in the dark. After three washes with serum-free culture medium, the cells were observed directly under a fluorescence microscope after loading the probe.

### 2.19. Transmission electron microscopy

Cells treated with BLM and Fostamatinib were harvested and immediately fixed in 2.5% glutaraldehyde overnight at 4 °C and post-fixed with 2% osmium tetroxide for 1 hour at 37 °C. Subsequently, cells were embedded and stained using uranylacetate/lead citrate. The sections were imaged using a transmission electron microscopy (TEM) (ThermoFisher Helios 5 UC).

### 2.20. Histological staining

Pulmonary tissues were collected from male C57BL/6 mice on day 20 after BLM or sterile saline treatment. The tissues were fixed in 4% paraformaldehyde, embedded in paraffin, and sectioned at 5 μm. Hematoxylin and eosin (H & E) (Solarbio), Masson's trichrome (Solarbio), and Sirius Red staining (Solarbio) were performed for histological evaluation.

### 2.21. CIBERSORT algorithm

Spearman’s rank correlation analysis was performed to evaluate the association between CHEK2 expression and the relative fractions of CIBERSORT-estimated immune cell types. The CIBERSORT output metrics, including P-value, correlation, and RMSE, were excluded from the immune-cell correlation analysis because they represent deconvolution quality metrics rather than immune cell fractions. To account for multiple testing across immune cell types, raw P values were adjusted using the Benjamini–Hochberg false discovery rate method. Correlations with an FDR-adjusted P value < 0.05 were considered statistically significant.

### 2.22. Statistical analysis

Data processing and analysis were performed using R software version 4.4.1. qRT-PCR data were analyzed with SPSS version 22 (IBM SPSS Statistics, USA). Statistical differences between experimental and control groups were assessed using an unpaired Student’s t-test and one-way ANOVA. A P value < 0.05 was considered statistically significant. Results are presented as mean ± standard deviation (SD).

### 2.23. Ethics statement

All experimental procedures were reviewed and approved by the Institutional Animal Care and Use Committee of Xiamen University (IACUC approval number: XMULAC20240170) and were conducted in accordance with the ARRIVE 2.0 guidelines and all relevant institutional and national regulations. All operators had undergone relevant training in animal experimental procedures. In addition, the data used in this study were mainly derived from public databases, and according to the requirements of the Ethics Committee of Zhongshan Hospital Affiliated to Xiamen University, the relevant ethical review was exempted.

## 3. Results

### 3.1. Identification and functional enrichment analysis of senescence-associated differentially expressed genes in idiopathic pulmonary fibrosis

**Fig 1:**
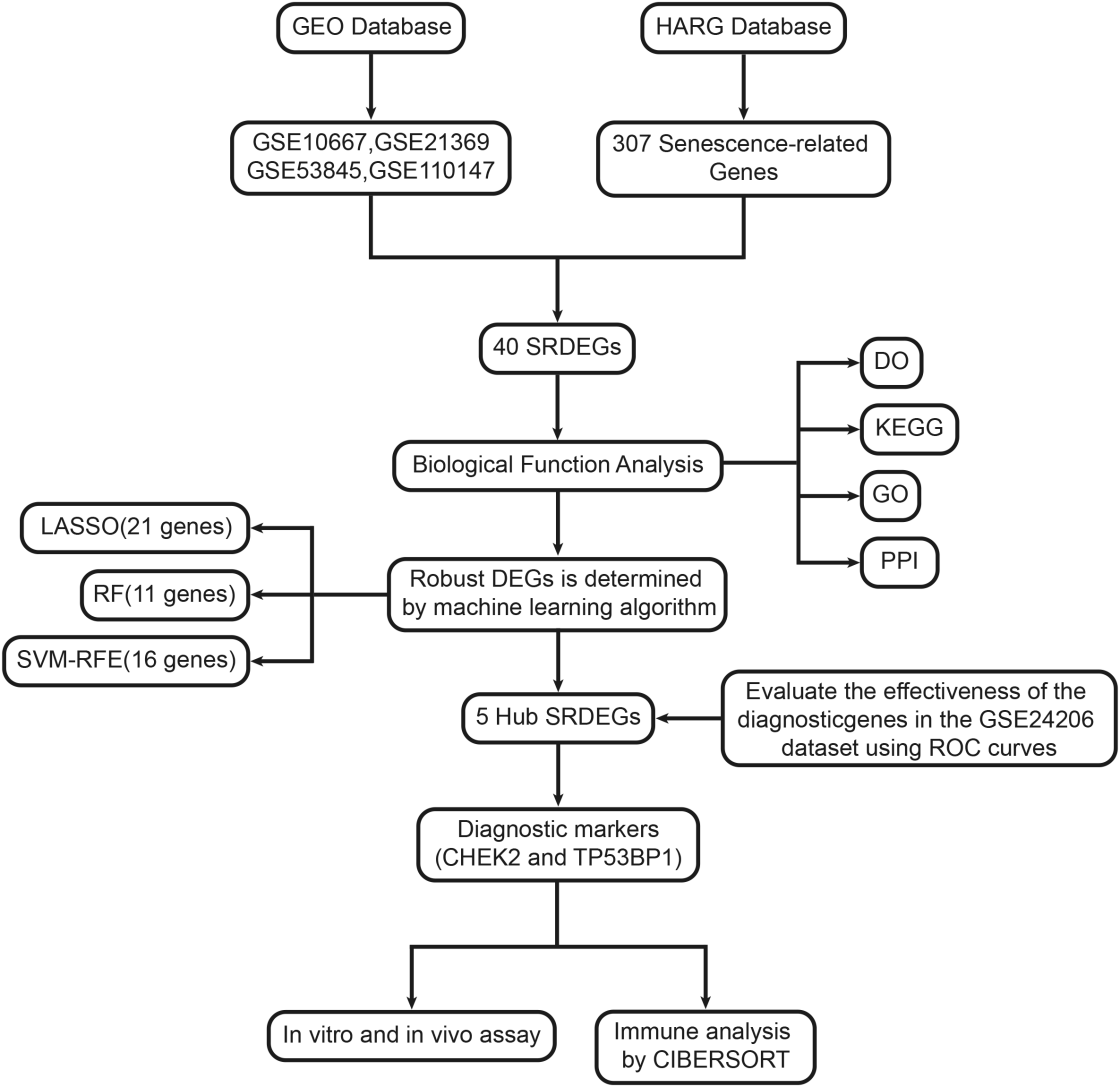
Overview of the study design.

**Fig 2:**
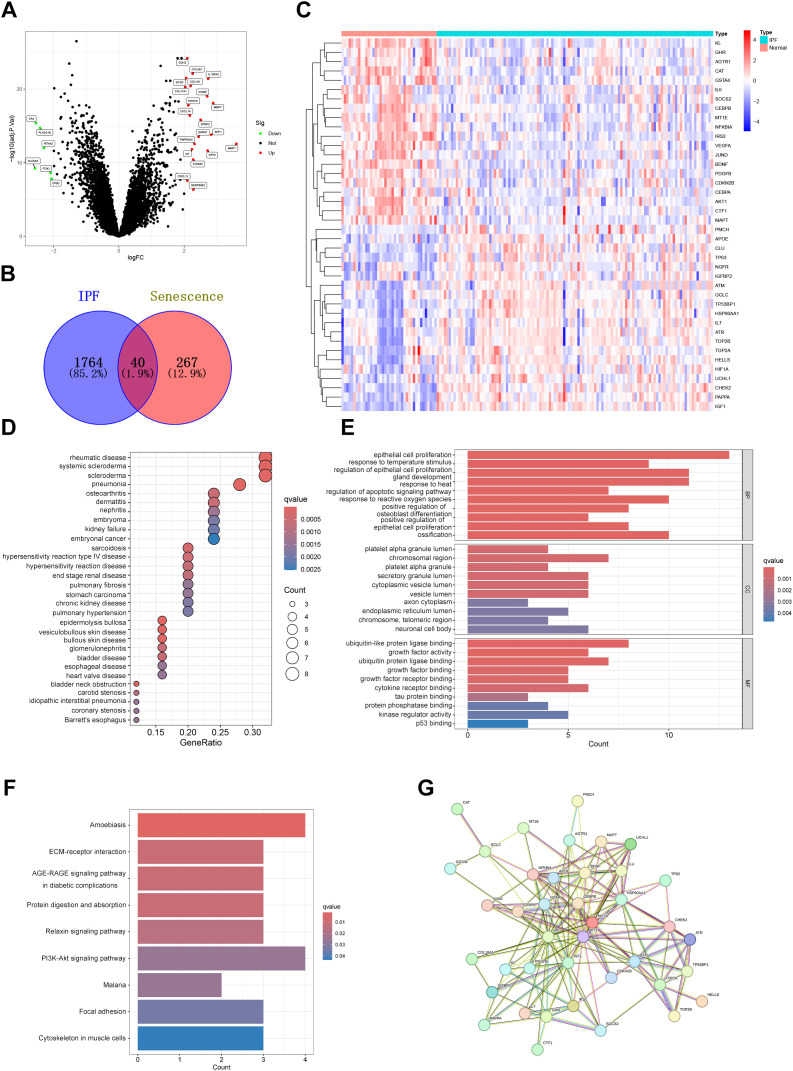
Identification and Functional Enrichment Analysis of Senescence-Associated Differentially Expressed Genes in Idiopathic Pulmonary Fibrosis. (A) Volcano plot of the GSE10667, GSE21369, GSE53845 and GSE110147 dataset with the cut-off criteria of |log2FC| >  0.5 and PFDR  <  0.05. (B) Venn diagram shows that 40 ARDEGs are identified from the intersection of genes between the IPF DEGs and HAGs. (C) Heatmap visualization of the ARDEGs between IPF and normal samples. (D) DO analysis of ARDEGs. (E) GO analysis of ARDEGs. (F) KEGG pathway enrichment analysis of ARDEGs. (G) PPI network and hub gene identification. PFDR<0.05.

The overall workflow of this study is illustrated in Fig 1. A total of 1,804 DEGs were identified between the normal control and IPF groups, including 942 upregulated and 862 downregulated genes (Fig 2A). By intersecting these DEGs with known human senescence -related genes, 40 SRDEGs were further screened (Fig 2B). The expression profiles of these SRDEGs are presented in a heatmap (Fig 2C).

To elucidate the biological significance of SRDEGs, systematic functional enrichment analyses were conducted, including DO, GO, KEGG, and PPI network analysis. DO analysis provided insights into potential pathological processes involving these genes. The results indicated that SRDEGs were significantly enriched in multiple age-related diseases, including rheumatoid arthritis, systemic sclerosis, scleroderma, pneumonia, and osteoarthritis (Fig 2D). These conditions are characterized by increased incidence or severity with senescence, suggesting a close association between cellular senescence and chronic inflammation, degenerative, or immune-mediated diseases. GO analysis further revealed the specific cellular functions in which these genes may participate in senescence phenotypes (Fig 2E). In terms of biological processes (BP), SRDEGs were mainly involved in epithelial cell proliferation, responses to temperature or heat stimuli, regulation of epithelial proliferation, and gland development—processes often impaired during tissue remodeling and age-related regenerative decline. Regarding cellular components (CC), SRDEGs were primarily localized to platelet α-granule lumen, chromosomal regions, secretory granule lumen, and cytoplasmic vesicle lumen, suggesting potential roles in stress response and intercellular signaling in senescent cells. Molecular function (MF) enrichment included ubiquitin-like protein ligase binding, growth factor activity, and receptor binding, which are closely related to proteostasis maintenance and growth factor signaling regulation, hallmark features of senescence. KEGG pathway analysis demonstrated that SRDEGs participated in multiple pathways closely linked to age-related pathologies (Fig 2F). Notably, the AGE-RAGE signaling pathway in diabetic complications was enriched, highlighting its relevance to vascular senescence and metabolic dysregulation. Additionally, SRDEGs were involved in relaxin signaling and protein digestion and absorption pathways, suggesting roles in metabolic regulation and extracellular matrix remodeling. To further investigate protein-level interactions, a PPI network comprising 40 nodes and 168 edges with an average node degree of 8.4 was constructed (Fig 2G). Network-level enrichment analysis revealed involvement in multiple senescence-associated BP, including positive regulation of osteoblast differentiation, peptide response, negative regulation of lipid localization, negative regulation of mitochondrial organization, and regulation of neuronal death. MF were enriched in ubiquitin-like protein ligase binding, protein dimerization activity, kinase binding, homodimerization activity, and histone deacetylase binding, suggesting a central role in proteostasis and epigenetic regulation. CC included enrichment in C/EBP complex, CHOP–C/EBP complex, neurofibrillary tangles, growth factor complex, and dendritic compartments, structures known to be altered during senescence and neurodegenerative conditions (S1 Table).

### 3.2. Construction of a risk model and diagnostic performance evaluation

**Fig 3:**
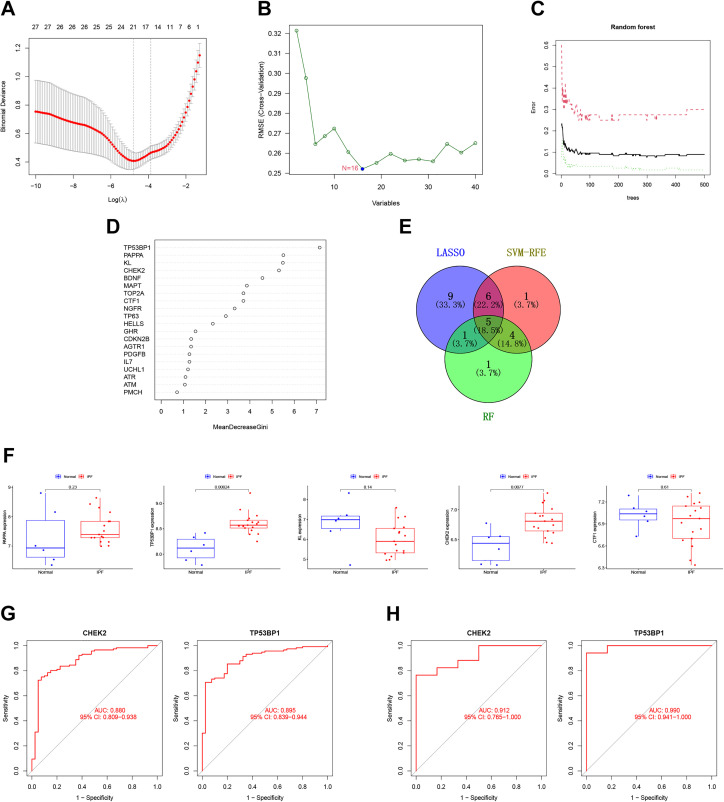
Construction of a Risk Model and Diagnostic Performance Evaluation. (A) The LASSO coefficient profiles of the 40 ARDEGs. (B) Estimating 10-fold cross-validation error using the SVM-RFE. (C) The relationship between the number of decision tree and the model error. (D) RF algorithm showed the top 20 candidate genes. (E) Venn diagram showed that five hub ARDEGs are identified via the above 3 algorithms. (F)Box plots of the expression of CHEK2 and TP53BP1 between normal and IPF samples in the training set. (G) ROC analysis was conducted for CHEK2 and TP53BP1 in training sets. (H) ROC analysis was conducted for CHEK2 and TP53BP1 in test sets. PFDR<0.05.

To develop a risk prediction model based on hub SRDEGs for precise IPF prediction, three machine learning algorithms (LASSO, SVM-RFE, and RF) were applied to the 40 SRDEGs to select candidate hub genes. LASSO identified 21 feature genes (Fig 3A), SVM-RFE identified 16 feature genes (Fig 3B), and RF identified 11 feature genes (Fig 3C-D). The intersection of the three algorithms yielded five hub SRDEGs (KL, TP53BP1, CHEK2, PAPPA, and CTF1) (Fig 3E). Boxplots confirmed the expression levels of these five hub genes (Fig 3F).

In the training dataset GSE24206, TP53BP1 and CHEK2 were significantly upregulated in IPF samples (p < 0.05). ROC curve analysis further evaluated their diagnostic performance, with CHEK2 achieving an AUC of 0.880 (95% CI: 0.809−0.938) and TP53BP1 an AUC of 0.895 (95% CI: 0.839−0.944) (Fig 3G). In an independent test set, CHEK2 and TP53BP1 demonstrated AUCs of 0.912 (95% CI: 0.765−1.000) and 0.990 (95% CI: 0.941−1.000), respectively (Fig 3H), indicating their robust ability to discriminate IPF from normal samples.

### 3.3. In vivo and in vitro validation of TP53BP1 and CHEK2

**Fig 4:**
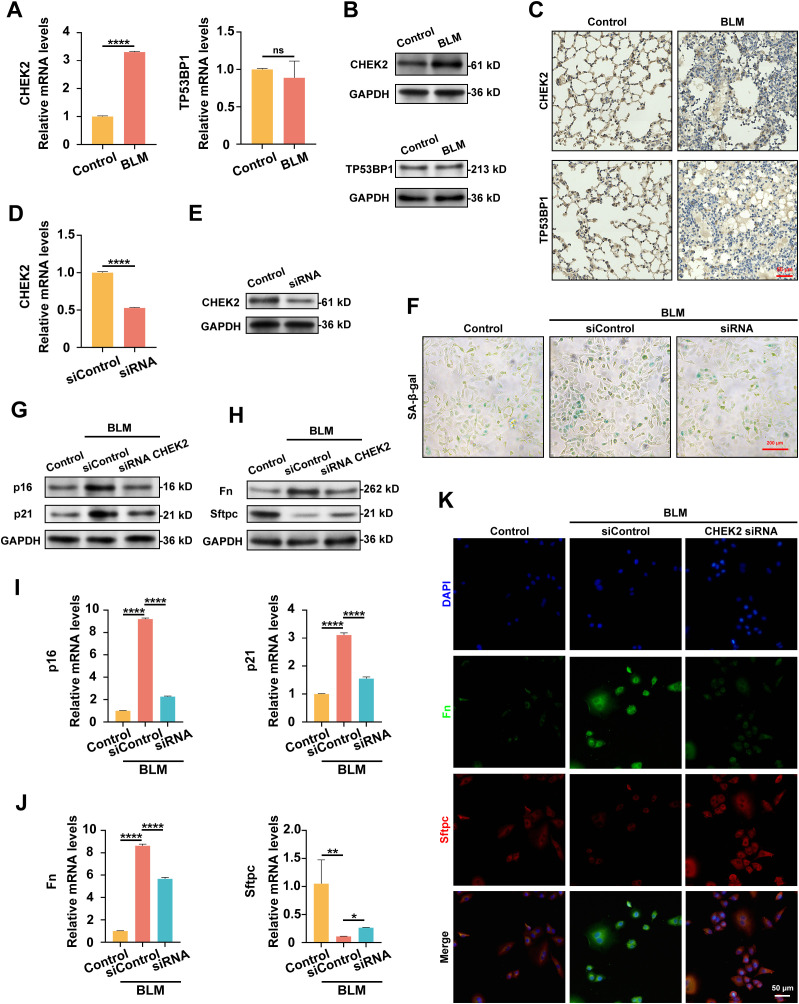
In Vivo and In Vitro Validation of TP53BP1 and CHEK2. (A) qRT-PCR analysis of CHEK2 and TP53BP1 mRNA levels in BLM-treated A549 cells. (B) Western blot analysis of CHEK2 and TP53BP1 protein ex-pression levels in BLM-treated A549 cells. (C) IHC analysis of CHEK2 and TP53BP1 expression levels in mouse lung tissue. (D) qRT-PCR analysis of CHEK2 mRNA levels in siRNA-treated A549 cells. (E) Western blot analysis of CHEK2 expression levels in siRNA-treated A549 cells. (F) SA-β-gal staining siRNA-treated A549 cells. (G) Western blot analysis of senescence markers p16 and p21 levels in siRNA-treated A549 cells. (H) Western blot analysis of EMT markers Sftpc and Fn levels in siRNA-treated A549 cells. (I) qRT-PCR analysis of p16 and p21 mRNA levels in BLM-treated A549 cells. (J) qRT-PCR analysis of Sftpc and Fn mRNA levels in BLM-treated A549 cells. (K) Immunofluorescence assay of EMT markers Sftpc and Fn levels in siRNA-treated A549 cells. Scale bars = 50 µm in (C), 200 µm in (F), 50 µm in (K). Data in (A, D, I and J) represent mean ± SD (n = 3, *P < 0.05, **P < 0.01, ***P < 0.001, ****P < 0.0001; ns indicates no significance; student’s t-test and one-way ANOVA).

To validate the above findings, we conducted additional experiments at the cellular and animal levels. qRT–PCR showed that CHEK2 mRNA was significantly upregulated in BLM-treated A549 cells, whereas TP53BP1 mRNA exhibited no significant change (Fig 4A). Western blot further confirmed increased CHEK2 protein abundance in BLM-induced A549 cells, while changes in TP53BP1 protein levels remained statistically non-significant (Fig 4B, S1 Fig A). Consistently, in the animal model, IHC staining revealed a marked increase in CHEK2 expression in fibrotic lung tissues, whereas no obvious increase was observed for TP53BP1 (Fig 4C). Collectively, these results suggest that among the candidate hub genes identified by machine learning, only CHEK2 demonstrates biological relevance; therefore, subsequent investigations were focused on CHEK2.

To explore the functional role of CHEK2 in IPF, we further performed siRNA-mediated knockdown (Fig 4D–E, S1 Fig B). Notably, SA-β-gal staining indicated that CHEK2 silencing significantly reduced the proportion of senescent A549 cells (Fig 4F, S2 Fig A). Meanwhile, both the transcript and protein levels of the senescence markers p16 and p21 were suppressed (Fig 4G, I, S1 Fig C). Given that EMT is a key pathological feature of IPF, BLM treatment induced upregulation of the mesenchymal marker Fn and downregulation of the epithelial marker Sftpc; importantly, these alterations were significantly reversed upon CHEK2 inhibition (Fig 4H, J, S1 Fig D). Co-localization assays of EMT-associated markers in A549 cells further supported these findings (Fig 4K). Taken together, these data indicate that CHEK2 may serve as a potential biomarker for IPF and plays a critical role in linking AEC senescence to EMT.

### 3.4. CHEK2-drug interaction analysis and molecular dynamics simulation

**Fig 5:**
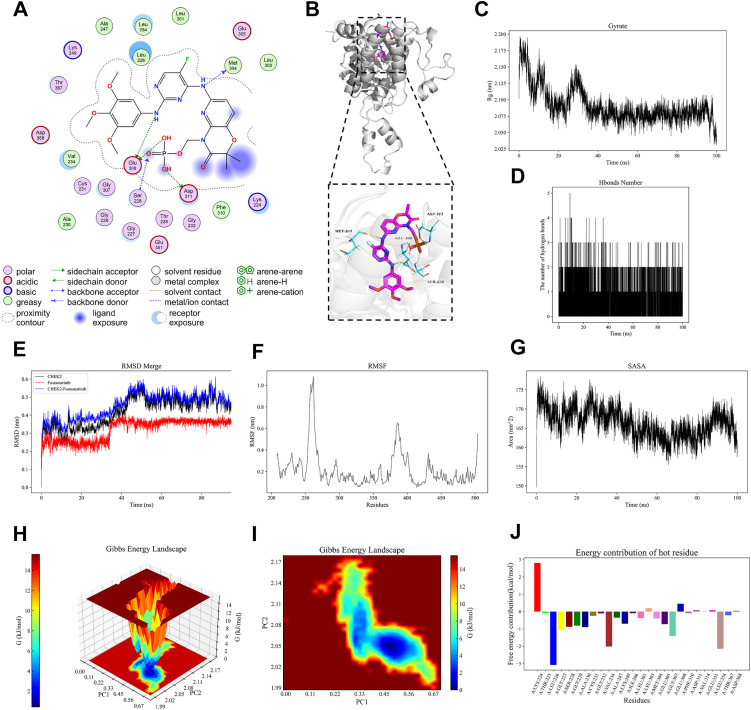
CHEK2-Drug Interaction Analysis and Molecular Dynamics Simulation. (A, B) Molecular docking analysis illustrating the predicted binding mode and interactions of FOS within the CHEK2 binding pocket. (C) Rg of the CHEK2 protein throughout the MD simulation trajectory. (D) Time-dependent variation in the number of hydrogen bonds formed between CHEK2 and FOS. (E) RMSD of the CHEK2 backbone (black), the FOS ligand (red), and the CHEK2-FOS complex (blue) over the simulation time. (F) RMSF of the FOS ligand atoms during the simulation. (G) SASA of the CHEK2-FOS complex. (H, I) 3D and 2D representations of the Gibbs free energy landscape for the CHEK2-FOS complex, respectively. (J) Per-residue binding free energy decomposition.

Building on CHEK2, the hub gene identified through machine learning and subsequent molecular validation, we further explored potential therapeutic intervention strategies. By screening the DrugBank database, we identified Fostamatinib as a putative CHEK2 inhibitor and predicted its molecular binding site. Molecular docking indicated a stable interaction between Fostamatinib and CHEK2, with a binding energy of −7.1 kcal/mol (S2 Tables). Specifically, Fostamatinib formed hydrogen bonds with residues MET-304, ASP-311, GLU-308, and SER-228, and engaged in hydrophobic interactions with surrounding amino acid residues (Fig 5A–B).

To further evaluate the structural stability, binding affinity, and dynamic features of the CHEK2–Fostamatinib complex, we performed MD simulations. Rg analysis suggested a compact overall protein conformation (mean, 2.09 ± 0.03 nm) without apparent unfolding events (Fig 5C). Hydrogen-bond analysis showed an average of 0.86 ± 0.67 hydrogen bonds, indicating a relatively stable anchoring effect (Fig 5D). RMSD values were low for the complex (0.46 ± 0.07 nm), ligand (0.32 ± 0.06 nm), and protein backbone (0.42 ± 0.08 nm), supporting high conformational stability (Fig 5E). RMSF was 0.21 ± 0.17 nm, further indicating limited overall flexibility, particularly within the binding region (Fig 5F). In addition, the SASA remained stable throughout the simulation (166.30 ± 3.67 nm²), consistent with the Rg and RMSD results (Fig 5G). The Gibbs free energy landscape revealed stable binding conformations, and MM/GBSA calculations yielded a total binding free energy (ΔTotal) of −27.25 ± 1.86 kcal/mol, with van der Waals interactions providing the largest contribution (ΔVDWAALS = −62.57 ± 0.78 kcal/mol) (Fig 5H–I). Residue decomposition analysis identified 26 key residues with substantial contributions to binding, among which LYS224 and LEU226 had the greatest impact (Fig 5J). Collectively, these results suggest strong binding affinity and favorable structural stability between Fostamatinib and CHEK2.

### 3.5. Fostamatinib attenuates IPF-associated cellular senescence and fibrosis via CHEK2 inhibition

**Fig 6:**
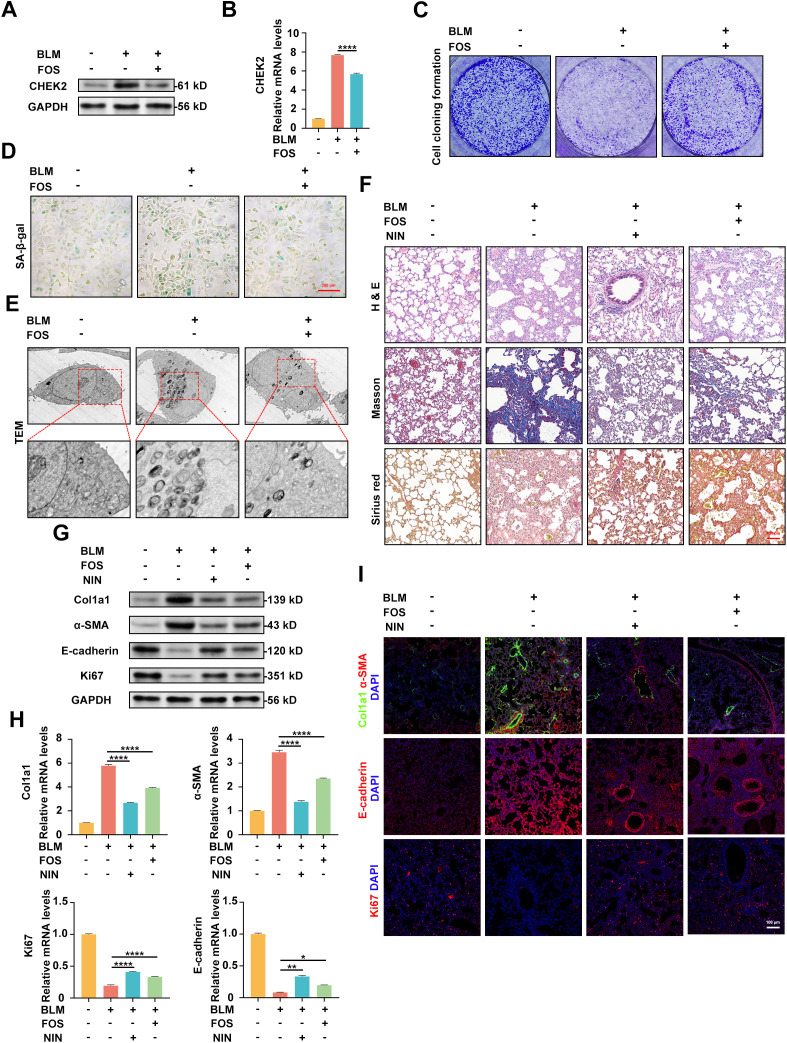
Fostamatinib Attenuates IPF-Associated Cellular Senescence and Fibrosis via CHEK2 Inhibition. (A) Western blot images of CHEK2 levels in A549 cells treated with FOS and BLM. (B) qRT-PCR analysis of CHEK2 mRNA levels in A549 cells. (C) Colony formation assay of A549 cells following treatment with FOS or BLM. (D) SA-β-gal staining A549 cells. (E) Representative TEM images showing autophagic vesicles in A549 cells. (F) Representative images of lung sections from mice treated with FOS or NIN after BLM challenge, stained with H&E, Masson's trichrome, and Sirius red. (G) Western blot images of Col1a1, α-SMA, Ki67 and E-cadherin levels in A549 cells treated with FOS and BLM. (H qRT-PCR analysis of Col1a1, α-SMA, Ki67 and E-cadherin mRNA levels in A549 cells. (I) Immunofluorescence staining of Col1a1, α-SMA, Ki67 and E-cadherin in mouse lung tissues. Scale bars = 200 µm in (D), 100 µm in (F, I). Data in (B, I) represent mean ± SD (n = 3, *P < 0.05, **P < 0.01, ***P < 0.001, ****P < 0.0001; ns indicates no significance; student’s t-test and one-way ANOVA).

We first determined the optimal concentration of Fostamatinib for treating A549 cells using a CCK-8 assay, which was 7.5 Μμ (S3 Fig). We then found that, in BLM-stimulated A549 cells, Fostamatinib reduced CHEK2 transcription and protein expression (Fig 6A, B, S1 Fig E). Because proliferative arrest and increased SA-β-gal staining are hallmark features of cellular senescence, we assessed these phenotypes and observed that Fostamatinib markedly alleviated both (Fig 6C–D, S2 Fig B). In addition, the accumulation of lipofuscin granules represents another key senescence-associated feature, and Fostamatinib treatment significantly decreased the intracellular levels of both (Fig 6F). In vivo, although Fostamatinib was slightly less effective than the positive-control drug nintedanib (NIN), it still substantially ameliorated BLM-induced disruption of alveolar architecture and fibrous tissue proliferation. To further delineate the effect of Fostamatinib on EMT, we measured the transcriptional and protein levels of fibrosis markers (Col1a1, α-SMA) and epithelial markers (Ki67 and E-cadherin) in lung tissues, and the results indicated that Fostamatinib improved EMT-related alterations to varying degrees (Fig 6H–J, S1 Fig F). Although the anti-fibrotic efficacy of Fostamatinib was modestly lower than that of NIN in our experiments, Fostamatinib remains of significant research interest as an agent that exerts anti-senescence effects by targeting CHEK2.

### 3.6. Immune cell infiltration and correlation analysis

**Fig 7:**
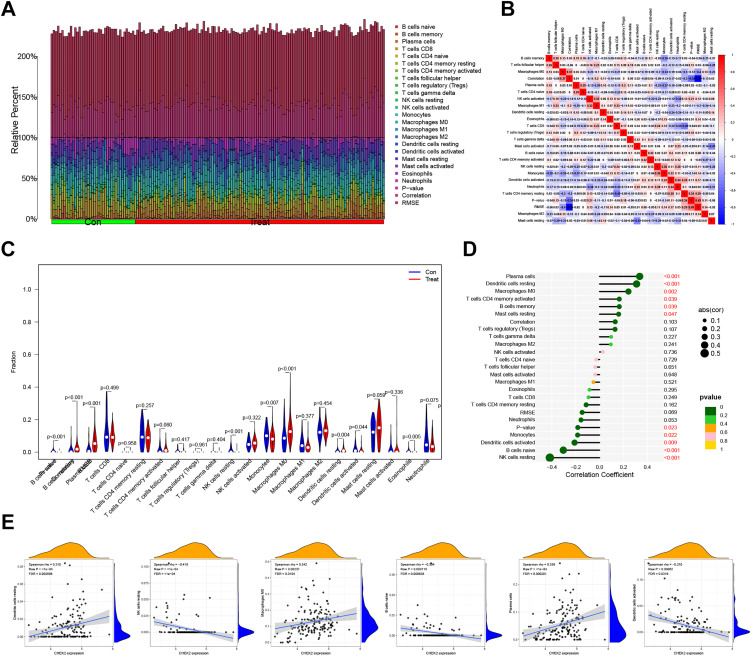
Immune Cell Infiltration and Correlation Analysis. (A) Bar plot of the percentage of the 25 types of immune cells between IPF group and control group. (B) Correlation of 25 types of immune cells. (C) Violin plot of the immune cell infiltration difference demonstrated that patients with idiopathic pulmonary fibrosis. (D-E) Correlation analysis between CHEK2 and immune cells.

IPF pathogenesis is closely associated with the immune microenvironment, shaped by chronic local inflammation and senescent cell interactions. Using deconvolution algorithms, we assessed immune cell infiltration in IPF samples. Immune cell infiltration between the IPF and control groups was assessed using the CIBERSORT algorithm. The percentage of 25 types of immune cells in IPF and control groups is shown in the bar plot (Fig 7A). Correlation analysis of these immune cells revealed that RMSE was negatively correlated with correlation (r = −0.99), and p-value was negatively correlated with correlation (r = −0.54). Conversely, RMSE was positively correlated with p-value (r = 0.49), and p-value was positively correlated with T cells CD4 memory resting (r = 0.34) (Fig 7B). The violin plot of immune cell infiltration demonstrated that IPF patients had higher levels of memory B cells, plasma cells, resting NK cells, and macrophages M0 compared to the control group (Fig 7C). Hub gene-immune cell correlation analysis indicated that CHEK2 had distinct associations with immune populations. CHEK2 expression correlated positively with M0 macrophages, resting dendritic cells, and plasma cells etc., but negatively with resting NK cells, naive B cells, and activated dendritic cells etc. (Fig 7D-7E). These findings reveal characteristic changes in the IPF immune microenvironment and suggest that CHEK2 may contribute to senescence-associated inflammation regulation, providing mechanistic insights into senescence -driven fibrosis.

## 4. Discussion

In this study, we integrated bioinformatics analyses, machine-learning algorithms, and experimental validation to systematically elucidate senescence – associated molecular mechanisms in IPF and to identify the key hub gene CHEK2. From the perspective of senescence – related genes, we comprehensively characterized the molecular features of IPF, offering critical insights into disease mechanisms and potential therapeutic targets.

We identified SRDEGs by intersecting DEGs between normal and IPF lung tissues with a senescence-related gene set. Functional enrichment analyses showed that these SRDEGs were associated with multiple canonical age-related diseases and were mainly involved in epithelial proliferation, gland development, proteostasis regulation, and AGE–RAGE signaling, suggesting that senescence-driven chronic inflammation, immune dysregulation, impaired regeneration, ECM remodeling, and metabolic imbalance may contribute to IPF progression [26,27]. Through integrated LASSO, SVM-RFE, and random forest analyses, CHEK2 and TP53BP1 were identified as hub SRDEGs with high diagnostic performance in both training and independent validation cohorts. Subsequent in vitro and in vivo validation revealed a more prominent differential expression pattern for CHEK2, which was therefore selected for further investigation. Functionally, CHEK2 silencing attenuated AEC senescence and reversed BLM-induced EMT, indicating that CHEK2 may act as both a potential biomarker and a key regulator of fibrotic progression in IPF.

CHEK2 is a key effector of the canonical DNA damage response checkpoint pathway [28]. Its upregulation in IPF may indicate increased epithelial genotoxic stress, including chronic DNA damage, replication stress, oxidative stress, or telomere-associated injury, which can activate downstream pathways such as p53/p21 and thereby promote persistent cell-cycle arrest and cellular senescence [29,30]. Although TP53BP1 did not show consistent changes in BLM-treated A549 cells or fibrotic mouse lung tissues, this finding does not exclude the involvement of TP53/p53-related signaling in IPF epithelial dysfunction. Previous single-cell studies have identified multiple abnormal epithelial populations in IPF lungs, including KRT8+ transitional cells with p53/NF-κB activation [31], senescent AT2 cells, KRT5−/KRT17+ pathologic epithelial cells, and aberrant basaloid cells enriched for senescence- and development-related programs [32,33]. One possible explanation is that TP53BP1 mainly acts as a DNA-damage scaffold protein, whose activity depends more on nuclear recruitment and post-translational regulation than on changes in total mRNA or protein abundance [34]. Similarly, the absence of a marked increase in total p53 activity markers in our A549 model may be related to the 72h sampling time after BLM exposure, transient or oscillatory p53 activation, phosphorylation- or localization-dependent regulation, feedback adaptation, and the limitations of cancer-derived A549 cells [35]. Therefore, the observed pattern of elevated CHEK2 with largely unchanged TP53BP1 may reflect preferential checkpoint activation and senescence maintenance rather than broad upregulation of DNA-repair scaffold factors. It should be noted that we did not observe a robust and sustained increase in p53 activity or expression at the late time points after bleomycin treatment in A549 cells [36,37]. This does not indicate a defective TP53 response in A549 cells (this cell line is TP53 wild-type and p53-responsive), but rather likely reflects the temporal characteristics of the experimental model. The p53 response to DNA damage is highly dynamic and can manifest as transient or pulsatile activation; the 72-hour time point may have missed the early activation peak. Moreover, low-to-moderate levels of DNA damage are more likely to induce pulsatile p53 activation and cell cycle arrest rather than sustained accumulation and apoptosis. Additionally, CHK2 can regulate p21 and senescence through both p53-dependent and p53-independent pathways. Therefore, our results only support that CHEK2-associated DNA damage/senescence signaling operates in bleomycin-treated A549 cells, and do not suggest that sustained p53 activation is the sole downstream mechanism.

The translational relevance of our study is exemplified by drug repurposing efforts, but the pharmacological interpretation of Fostamatinib requires caution. Fostamatinib is an orally available prodrug of R406 and is classically recognized as a relatively selective SYK inhibitor [38]. R406 has also been reported to exert senolytic activity in senescent cells through pathways including FAK and p38 MAPK, and SYK inhibition has been linked to attenuation of lung injury and fibrosis-associated inflammatory signaling [39]. Therefore, although our docking and MD simulations predicted a stable interaction between Fostamatinib and CHEK2 and our experiments showed reduced CHEK2 expression and attenuation of senescence/fibrotic phenotypes after Fostamatinib treatment, these findings do not prove that CHEK2 is the sole direct pharmacological target responsible for the observed effects. Rather, Fostamatinib may act through CHEK2-associated DNA-damage/senescence signaling, SYK-dependent inflammatory or senescence pathways, or a combined mechanism. Future studies using direct CHEK2 kinase activity assays, CHEK2 rescue experiments, CRISPR-mediated CHEK2 knockout, and primary AT2 or organoid models will be required to determine the extent to which Fostamatinib acts through CHEK2-specific inhibition.

Our study also suggests potential interactions between senescence-associated pathways and the IPF immune microenvironment. Immune cells, particularly macrophages and T cells, play essential roles in fibrosis progression [40]. Dysregulated ARGs may affect immune cell behavior, exacerbating inflammation and fibrotic processes. Future studies should focus on delineating the mechanistic crosstalk between senescent cells and the immune microenvironment in IPF to uncover novel therapeutic targets.

Despite the insights provided, our study has limitations. First, the use of public datasets may introduce biases due to differences in sample collection and processing. Second, although A549 cells are convenient and widely used for BLM-induced epithelial injury and senescence studies, they are adenocarcinoma-derived and do not fully model primary AT2-cell biology, validation in primary human AT2 cells, induced pluripotent stem cell-derived alveolar organoids, or precision-cut lung slices is needed. Third, our biomarker analysis was primarily tissue-based, and whether CHEK2 can serve as a clinically feasible blood-based biomarker remains unknown. Future research should incorporate larger independent cohorts, blood/BALF validation, primary AT2-cell or organoid models, direct kinase assays, and single-cell or spatial validation to clarify clinical applicability and therapeutic potential.

## 5. Conclusion

This study systematically delineates the senescence-associated molecular features of IPF and identifies CHEK2 and TP53BP1 as key hub genes. CHEK2, as an upstream kinase of the p53 signaling pathway, cooperates with TP53BP1 to drive AECs senescence and EMT, promoting IPF onset and progression. We further demonstrated that Fostamatinib, a clinically approved drug, can stably bind and effectively inhibit CHEK2 activity, mitigating cellular senescence and fibrosis. Immune infiltration analysis revealed that CHEK2 and TP53BP1 expression is closely associated with alterations in immune cell populations, suggesting that the interaction between senescence and the immune microenvironment is a critical mechanism in IPF pathogenesis. This study not only deepens understanding of senescence-related molecular mechanisms in IPF but also provides a solid experimental and theoretical basis for CHEK2-targeted interventions. The drug repurposing strategy for Fostamatinib offers a feasible pathway for developing novel anti-IPF therapies, paving the way for future precision medicine approaches and clinical translation.

## Supporting information

S1 Fig The relative protein levels were normalized to GAPDH and calculated as fold change relative to the control group. Data in figure represent mean ± SD (n = 3, *P < 0.05, **P < 0.01, ***P < 0.001, ****P < 0.0001; ns indicates no significance; student’s t-test and one-way ANOVA).
(TIF)

S2 Fig Normalized quantitative analysis of SA-β-gal positive cell ratio. Data in figure represent mean ± SD (n = 3, *P < 0.05, **P < 0.01, ***P < 0.001, ****P < 0.0001; ns indicates no significance; student’s t-test and one-way ANOVA).
(TIF)

S3 Fig CCK8 results of A549 cells treated with different concentrations of SAL for 24 hours. Data in figure represent mean ± SD (n = 3, *P < 0.05, **P < 0.01, ***P < 0.001, ****P < 0.0001; ns indicates no significance; student’s t-test).
(TIF)

S1 Table GO and KEGG of Charts of GO and KEGG analysis of PPI – related interacting proteins. (DOCX)

S2 Table Molecular docking results and scoring. (DOCX)

S1 Raw Westernblot raw materials. (PDF)

S2 Raw RT-qPCR raw materials. (XLSX)

## References

[1] Mora AL, Rojas M, Pardo A, Selman M. Emerging therapies for idiopathic pulmonary fibrosis, a progressive age-related disease. Nat Rev Drug Discov. 2017;16(11):755–72. doi: 10.1038/nrd.2017.170 28983101

[2] Merkt W, Rodon L, Deicher FS, Freitag M, Claus M, Lister R, et al. Natural killer cell immunotherapy reverses lung fibrosis by eliminating senescent fibroblasts. Sci Transl Med. 2026;18(849):eadq5442. doi: 10.1126/scitranslmed.adq5442 42127218

[3] Maher TM, Bendstrup E, Dron L, Langley J, Smith G, Khalid JM, et al. Global incidence and prevalence of idiopathic pulmonary fibrosis. Respir Res. 2021;22(1):197. doi: 10.1186/s12931-021-01791-z 34233665 PMC8261998

[4] TM M. Interstitial lung disease: a review. JAMA. 2024;331(19):1655–65. doi: 10.1001/jama.2024.3669 38648021

[5] Chambers DC, Cherikh WS, Harhay MO, Hayes D Jr, Hsich E, Khush KK, et al. The International Thoracic Organ Transplant Registry of the International Society for Heart and Lung Transplantation: Thirty-sixth adult lung and heart-lung transplantation Report-2019; Focus theme: Donor and recipient size match. J Heart Lung Transplant. 2019;38(10):1042–55. doi: 10.1016/j.healun.2019.08.001 31548030 PMC6816340

[6] Patel H, Shah JR, Patel DR, Avanthika C, Jhaveri S, Gor K. Idiopathic pulmonary fibrosis: Diagnosis, biomarkers and newer treatment protocols. Dis Mon. 2023;69(7):101484. doi: 10.1016/j.disamonth.2022.101484 36220705

[7] Zhu W, Liu C, Tan C, Zhang J. Predictive biomarkers of disease progression in idiopathic pulmonary fibrosis. Heliyon. 2024;10(1):e23543. doi: 10.1016/j.heliyon.2023.e23543 38173501 PMC10761784

[8] Yao C, Guan X, Carraro G, Parimon T, Liu X, Huang G, et al. Senescence of Alveolar Type 2 Cells Drives Progressive Pulmonary Fibrosis. Am J Respir Crit Care Med. 2021;203(6):707–17. doi: 10.1164/rccm.202004-1274OC 32991815 PMC7958503

[9] Wolters PJ, Blackwell TS, Eickelberg O, Loyd JE, Kaminski N, Jenkins G, et al. Time for a change: is idiopathic pulmonary fibrosis still idiopathic and only fibrotic? Lancet Respir Med. 2018;6(2):154–60. doi: 10.1016/S2213-2600(18)30007-9 29413083 PMC5903445

[10] He Y, Thummuri D, Zheng G, Okunieff P, Citrin DE, Vujaskovic Z, et al. Cellular senescence and radiation-induced pulmonary fibrosis. Transl Res. 2019;209:14–21. doi: 10.1016/j.trsl.2019.03.006 30981698 PMC6857805

[11] Waters DW, Blokland KEC, Pathinayake PS, Burgess JK, Mutsaers SE, Prele CM, et al. Fibroblast senescence in the pathology of idiopathic pulmonary fibrosis. Am J Physiol Lung Cell Mol Physiol. 2018;315(2):L162–72. doi: 10.1152/ajplung.00037.2018 29696986 PMC6139657

[12] Wang Q, Xie Z-L, Wu Q, Jin Z-X, Yang C, Feng J. Role of various imbalances centered on alveolar epithelial cell/fibroblast apoptosis imbalance in the pathogenesis of idiopathic pulmonary fibrosis. Chin Med J (Engl). 2021;134(3):261–74. doi: 10.1097/CM9.0000000000001288 33522725 PMC7846426

[13] Sun W, Jing X, Yang X, Huang H, Luo Q, Xia S, et al. Regulation of the IGF1 signaling pathway is involved in idiopathic pulmonary fibrosis induced by alveolar epithelial cell senescence and core fucosylation. Aging (Albany NY). 2021;13(14):18852–69. doi: 10.18632/aging.203335 34329195 PMC8351684

[14] Lehmann M, Korfei M, Mutze K, Klee S, Skronska-Wasek W, Alsafadi HN, et al. Senolytic drugs target alveolar epithelial cell function and attenuate experimental lung fibrosis ex vivo. Eur Respir J. 2017;50(2). doi: 10.1183/13993003.02367-2016 PMC5593348 28775044

[15] Sato S, Koyama K, Ogawa H, Murakami K, Imakura T, Yamashita Y, et al. A novel BRD4 degrader, ARV-825, attenuates lung fibrosis through senolysis and antifibrotic effect. Respir Investig. 2023;61(6):781–92. doi: 10.1016/j.resinv.2023.08.003 37741093

[16] Linge A, Weinhold K, Bläsche R, Kasper M, Barth K. Downregulation of caveolin-1 affects bleomycin-induced growth arrest and cellular senescence in A549 cells. Int J Biochem Cell Biol. 2007;39(10):1964–74. doi: 10.1016/j.biocel.2007.05.018 17662641

[17] Haug CJ, Drazen JM. Artificial intelligence and machine learning in clinical medicine. N Engl J Med. 2023;388(13):1201–8. doi: 10.1056/NEJMra2302038 36988595

[18] Forrest IS, Petrazzini BO, Duffy Á, Park JK, Marquez-Luna C, Jordan DM, et al. Machine learning-based marker for coronary artery disease: derivation and validation in two longitudinal cohorts. Lancet. 2023;401(10372):215–25. doi: 10.1016/S0140-6736(22)02079-7 36563696 PMC10069625

[19] McIntosh C, Conroy L, Tjong MC, Craig T, Bayley A, Catton C, et al. Clinical integration of machine learning for curative-intent radiation treatment of patients with prostate cancer. Nat Med. 2021;27(6):999–1005. doi: 10.1038/s41591-021-01359-w 34083812

[20] Bartek J, Lukas J. Chk1 and Chk2 kinases in checkpoint control and cancer. Cancer Cell. 2003;3(5):421–9. doi: 10.1016/s1535-6108(03)00110-7 12781359

[21] Rossi DJ, Bryder D, Seita J, Nussenzweig A, Hoeijmakers J, Weissman IL. Deficiencies in DNA damage repair limit the function of haematopoietic stem cells with age. Nature. 2007;447(7145):725–9. doi: 10.1038/nature05862 17554309

[22] Stubbs FE, Flynn BP, Rivers CA, Birnie MT, Herman A, Swinstead EE, et al. Identification of a novel GR-ARID1a-P53BP1 protein complex involved in DNA damage repair and cell cycle regulation. Oncogene. 2022;41(50):5347–60. doi: 10.1038/s41388-022-02516-2 36344675 PMC9734058

[23] Di Micco R, Sulli G, Dobreva M, Liontos M, Botrugno OA, Gargiulo G, et al. Interplay between oncogene-induced DNA damage response and heterochromatin in senescence and cancer. Nat Cell Biol. 2011;13(3):292–302. doi: 10.1038/ncb2170 21336312 PMC3918344

[24] Rodier F, Campisi J. Four faces of cellular senescence. J Cell Biol. 2011;192(4):547–56. doi: 10.1083/jcb.201009094 21321098 PMC3044123

[25] Wu X, Wei J, Ran W, Liu D, Yi Y, Gong M, et al. The Gut Microbiota-Xanthurenic Acid-Aromatic Hydrocarbon Receptor Axis Mediates the Anticolitic Effects of Trilobatin. Adv Sci (Weinh). 2025;12(10):e2412234. doi: 10.1002/advs.202412234 39836604 PMC11904984

[26] Moss BJ, Ryter SW, Rosas IO. Pathogenic Mechanisms Underlying Idiopathic Pulmonary Fibrosis. Annu Rev Pathol. 2022;17:515–46. doi: 10.1146/annurev-pathol-042320-030240 34813355

[27] DePianto DJ, Heiden JAV, Morshead KB, Sun K-H, Modrusan Z, Teng G, et al. Molecular mapping of interstitial lung disease reveals a phenotypically distinct senescent basal epithelial cell population. JCI Insight. 2021;6(8):e143626. doi: 10.1172/jci.insight.143626 33705361 PMC8119199

[28] Smith HL, Southgate H, Tweddle DA, Curtin NJ. DNA damage checkpoint kinases in cancer. Expert Rev Mol Med. 2020;22:e2. doi: 10.1017/erm.2020.3 32508294

[29] Schuliga M, Kanwal A, Read J, Blokland KEC, Burgess JK, Prêle CM, et al. A cGAS-dependent response links DNA damage and senescence in alveolar epithelial cells: a potential drug target in IPF. Am J Physiol Lung Cell Mol Physiol. 2021;321(5):L859–71. doi: 10.1152/ajplung.00574.2020 34524912

[30] Rass E, Willaume S, Bertrand P. 53BP1: Keeping It under Control, Even at a Distance from DNA Damage. Genes (Basel). 2022;13(12):2390. doi: 10.3390/genes13122390 36553657 PMC9778356

[31] Strunz M, Simon LM, Ansari M, Kathiriya JJ, Angelidis I, Mayr CH, et al. Alveolar regeneration through a Krt8+ transitional stem cell state that persists in human lung fibrosis. Nat Commun. 2020;11(1):3559. doi: 10.1038/s41467-020-17358-3 32678092 PMC7366678

[32] García-Santisteban I, Llopis A, Krenning L, Vallejo-Rodríguez J, van den Broek B, Zubiaga AM, et al. Sustained CHK2 activity, but not ATM activity, is critical to maintain a G1 arrest after DNA damage in untransformed cells. BMC Biol. 2021;19(1):35. doi: 10.1186/s12915-021-00965-x 33607997 PMC7896382

[33] Habermann AC, Gutierrez AJ, Bui LT, Yahn SL, Winters NI, Calvi CL, et al. Single-cell RNA sequencing reveals profibrotic roles of distinct epithelial and mesenchymal lineages in pulmonary fibrosis. Sci Adv. 2020;6(28):eaba1972. doi: 10.1126/sciadv.aba1972 32832598 PMC7439444

[34] Wang X-L, Xu Y-T, Zhang S-L, Zhu X-Y, Zhang H-X, Liu Y-J. Hydrogen sulfide inhibits alveolar type II cell senescence and limits pulmonary fibrosis via promoting MDM2-mediated p53 degradation. Acta Physiol (Oxf). 2024;240(1):e14059. doi: 10.1111/apha.14059 37987182

[35] Lou J, Priest DG, Solano A, Kerjouan A, Hinde E. Spatiotemporal dynamics of 53BP1 dimer recruitment to a DNA double strand break. Nat Commun. 2020;11(1):5776. doi: 10.1038/s41467-020-19504-3 33188174 PMC7666136

[36] Rana T, Jiang C, Banerjee S, Yi N, Zmijewski JW, Liu G, et al. PAI-1 Regulation of p53 Expression and Senescence in Type II Alveolar Epithelial Cells. Cells. 2023;12(15). doi: 10.3390/cells12152008 PMC10417428 37566086

[37] Tsabar M, Mock CS, Venkatachalam V, Reyes J, Karhohs KW, Oliver TG, et al. A Switch in p53 Dynamics Marks Cells That Escape from DSB-Induced Cell Cycle Arrest. Cell Rep. 2020;32(5):107995. doi: 10.1016/j.celrep.2020.107995 32755587 PMC7521664

[38] Matsukane R, Suetsugu K, Hirota T, Ieiri I. Clinical Pharmacokinetics and Pharmacodynamics of Fostamatinib and Its Active Moiety R406. Clin Pharmacokinet. 2022;61(7):955–72. doi: 10.1007/s40262-022-01135-0 35781630 PMC9250994

[39] Kost-Alimova M, Sidhom E-H, Satyam A, Chamberlain BT, Dvela-Levitt M, Melanson M, et al. A High-Content Screen for Mucin-1-Reducing Compounds Identifies Fostamatinib as a Candidate for Rapid Repurposing for Acute Lung Injury. Cell Rep Med. 2020;1(8):100137. doi: 10.1016/j.xcrm.2020.100137 33294858 PMC7691435

[40] Serezani APM, Pascoalino BD, Bazzano JMR, Vowell KN, Tanjore H, Taylor CJ, et al. Multiplatform Single-Cell Analysis Identifies Immune Cell Types Enhanced in Pulmonary Fibrosis. Am J Respir Cell Mol Biol. 2022;67(1):50–60. doi: 10.1165/rcmb.2021-0418OC 35468042 PMC9273229

