# Supplementary material for: Identification of senescence-related genes in diagnosing idiopathic pulmonary fibrosis via integrating bioinformatics analysis and machine learning
Source: PLoS One. 2026 Aug 7;21(8):e0353694. doi: 10.1371/journal.pone.0353694 (PMC13450802; doi:10.1371/journal.pone.0353694)

Fig4 B

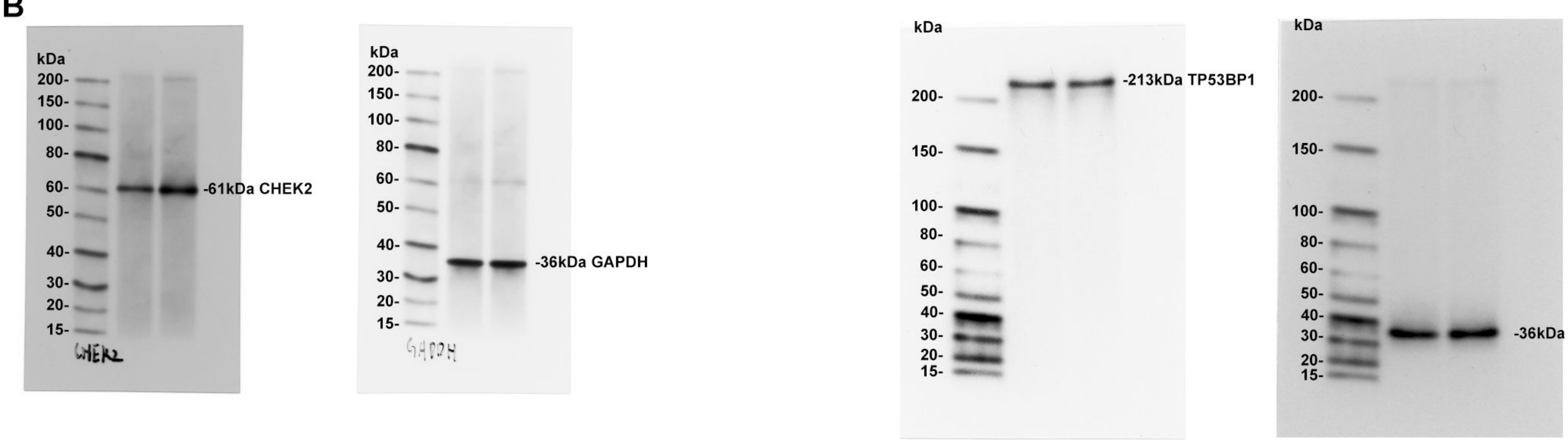

Fig4 E

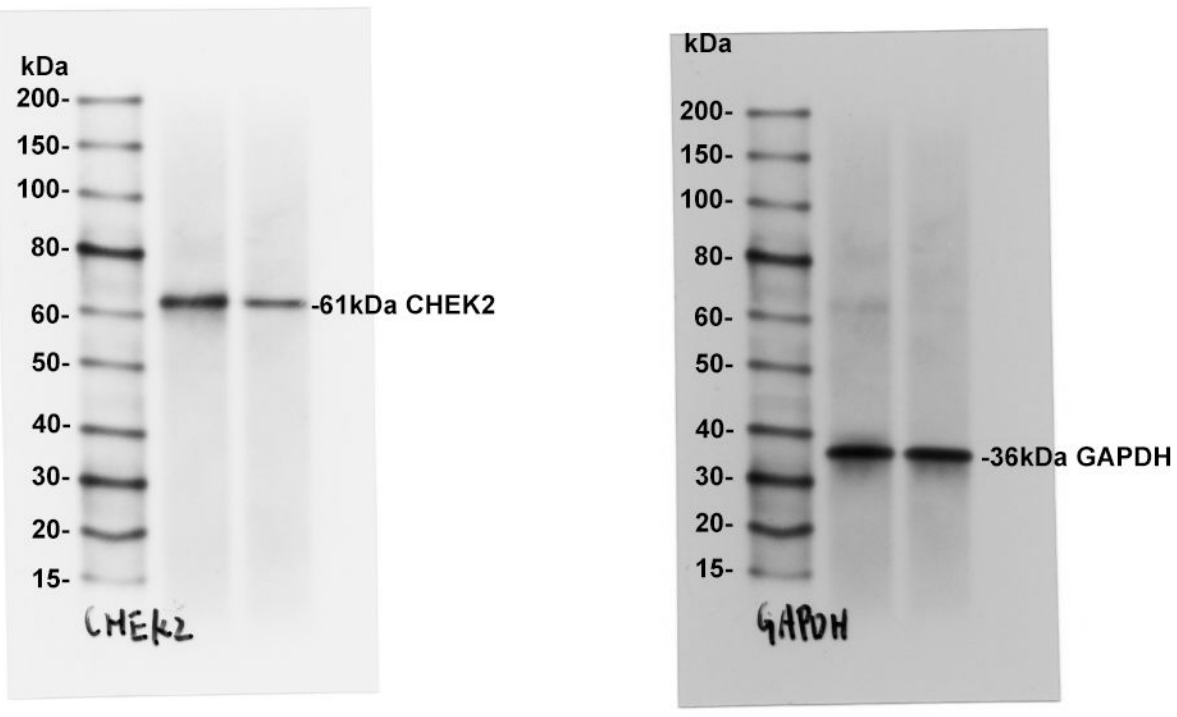

Fig4 G

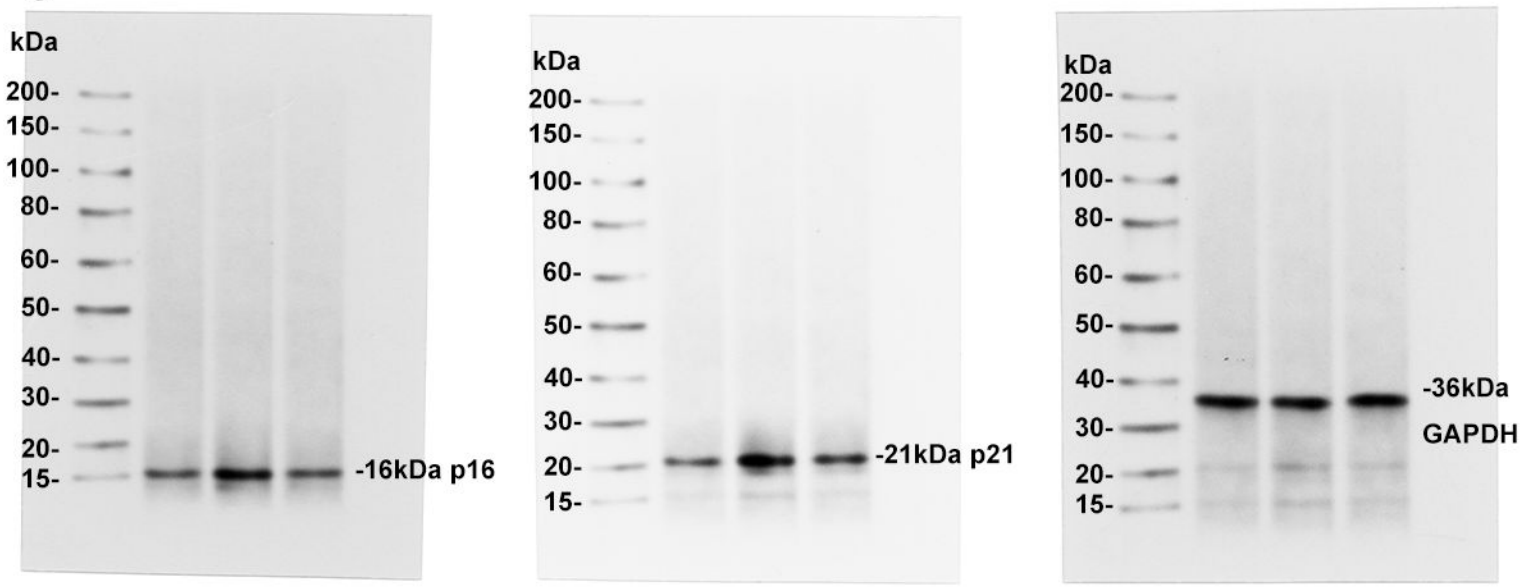

Fig4 H

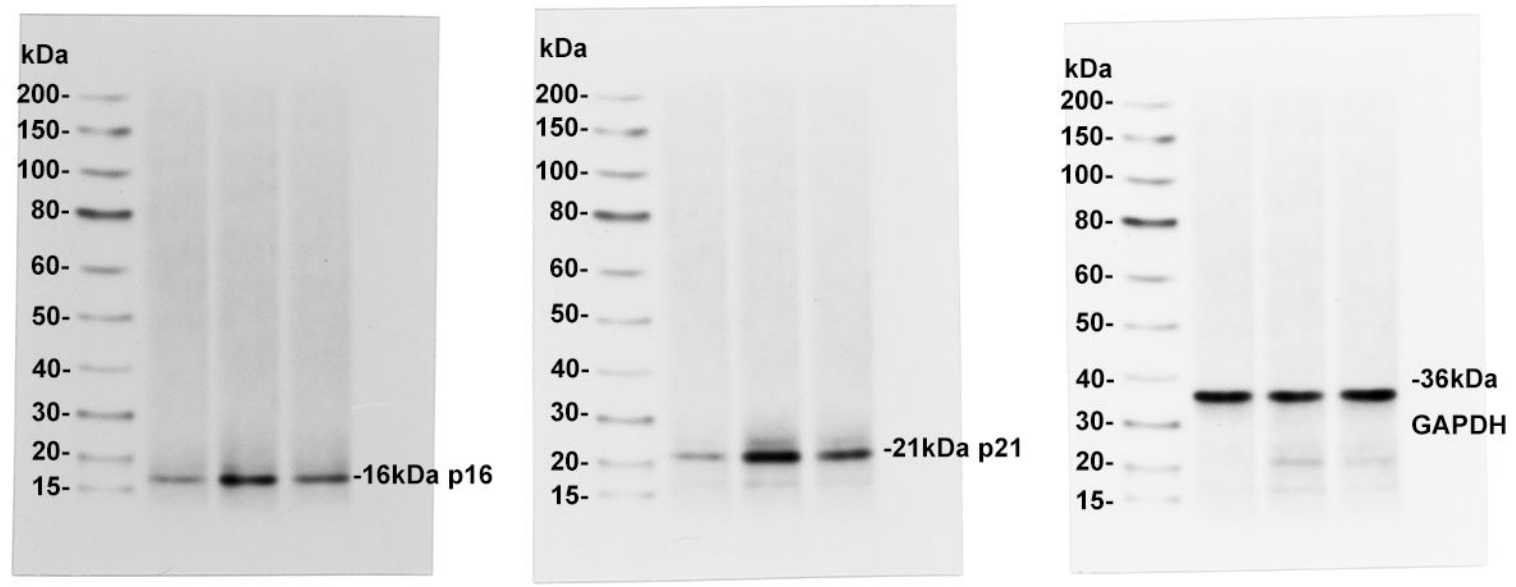

Fig4 I

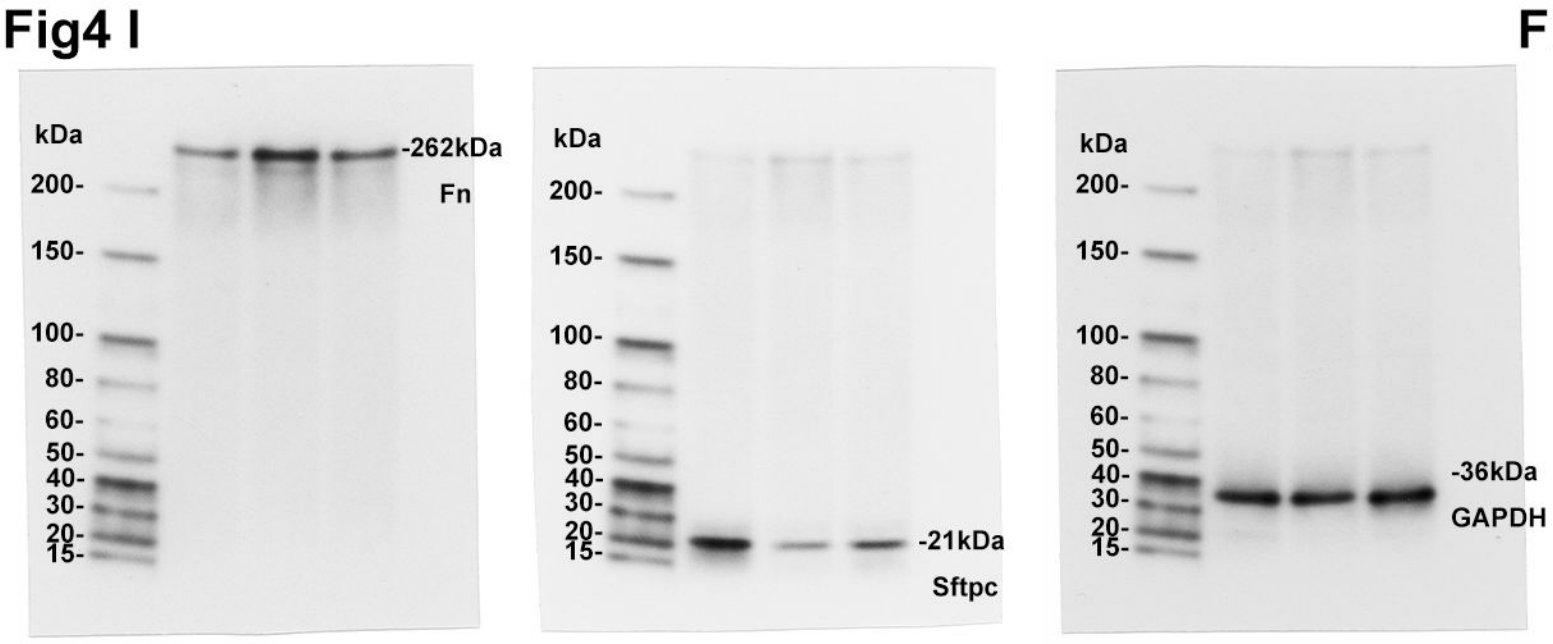

Fig4 J

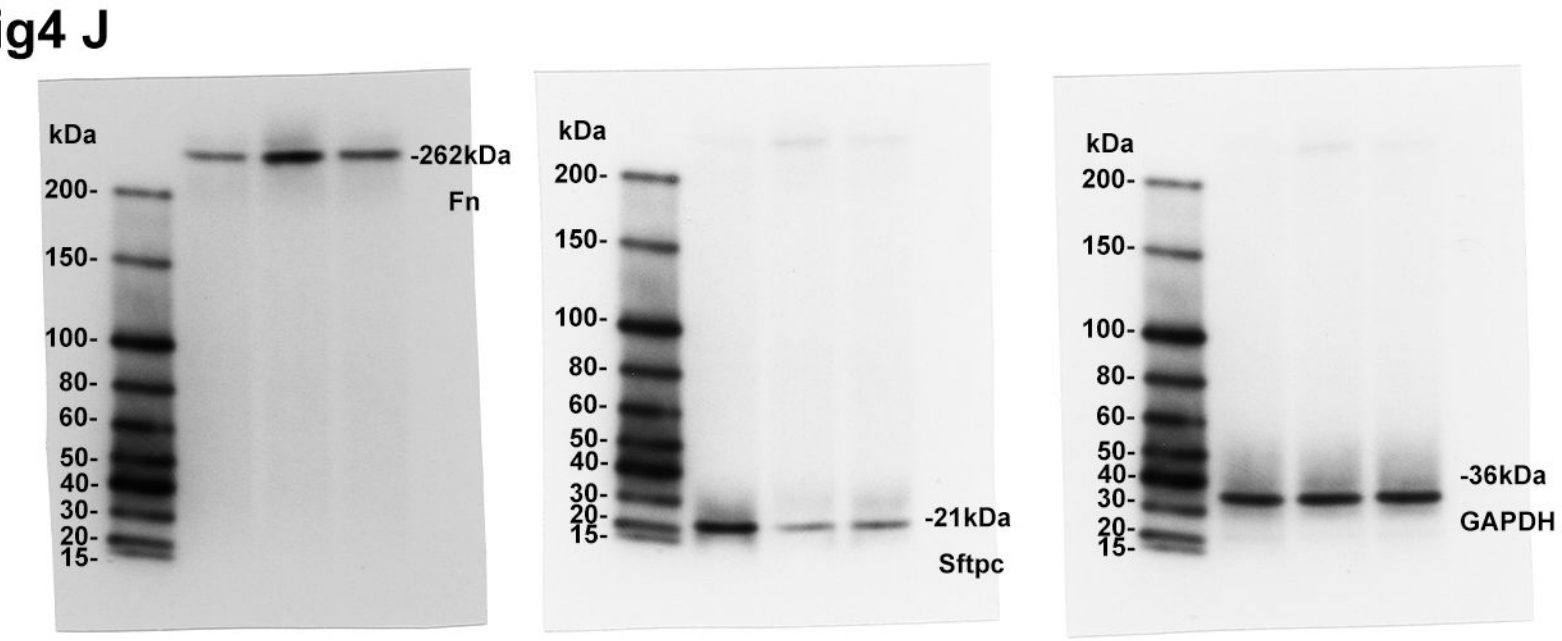

Fig6 A

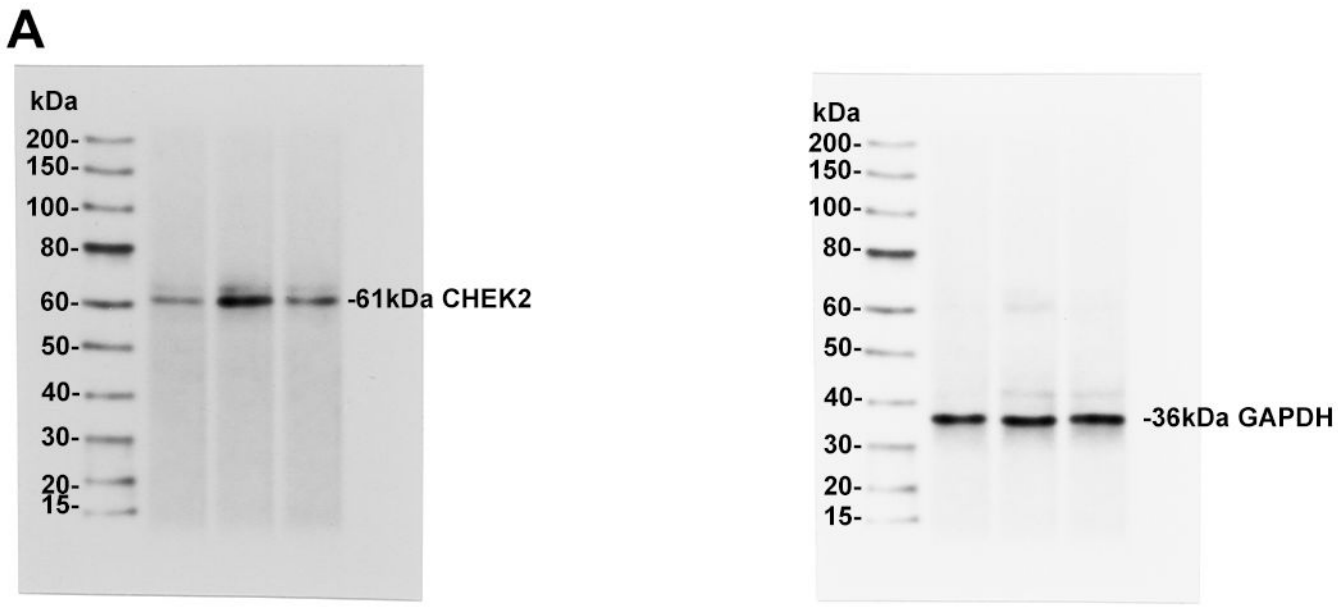

Fig6 H

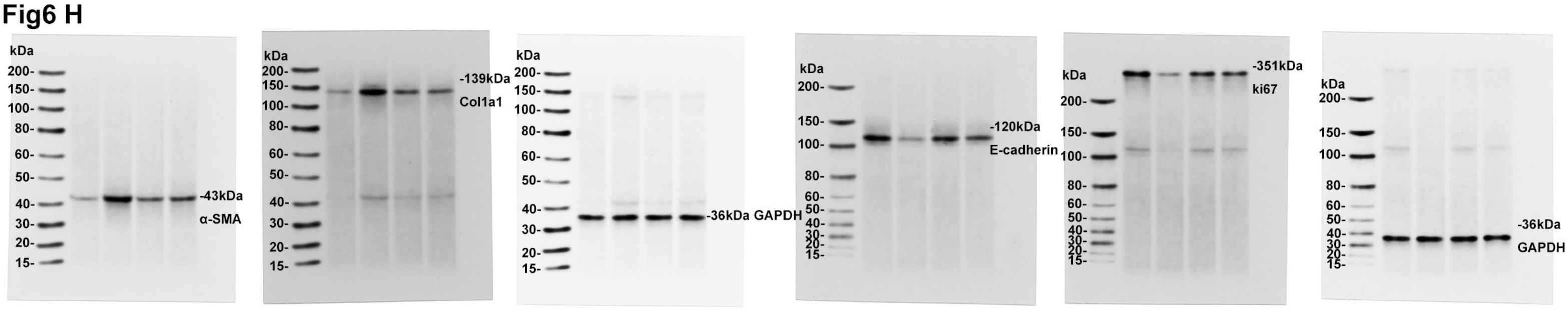

Supplement: Supplementary file 1 [file pone.0353694.s006.pdf]
