# Supplementary material for: Identification of senescence-related genes in diagnosing idiopathic pulmonary fibrosis via integrating bioinformatics analysis and machine learning
Source: PLoS One. 2026 Aug 7;21(8):e0353694. doi: 10.1371/journal.pone.0353694 (PMC13450802; doi:10.1371/journal.pone.0353694)
Supplement: Supplementary file 2 [file pone.0353694.s004.docx]

**Biological Process**

| **GO - term** | **description** | **count in network** | **strength** | **signal** | **false discovery rate** |
| --- | --- | --- | --- | --- | --- |
| GO:0045669 | Positive regulation of osteoblast differentiation | 6 of 68 | 1.64 | 1.86 | 1.34e - 06 |
| GO:1901652 | Response to peptide | 13 of 437 | 1.17 | 1.76 | 1.77e - 09 |
| GO:1905953 | Negative regulation of lipid localization | 5 of 47 | 1.72 | 1.68 | 8.57e - 06 |
| GO:0010823 | Negative regulation of mitochondrion organization | 5 of 54 | 1.66 | 1.58 | 1.52e - 05 |
| GO:1901214 | Regulation of neuron death | 10 of 324 | 1.18 | 1.52 | 2.34e - 07 |

**Molecular Function (Gene Ontology)**

| **GO - term** | **description** | **count in network** | **strength** | **signal** | **false discovery rate** |
| --- | --- | --- | --- | --- | --- |
| GO:0044389 | Ubiquitin - like protein ligase binding | 8 of 317 | 1.09 | 0.99 | 0.00015 |
| GO:0046983 | Protein dimerization activity | 15 of 1094 | 0.83 | 0.93 | 4.20e - 06 |
| GO:0019900 | Kinase binding | 12 of 785 | 0.88 | 0.9 | 3.36e - 05 |
| GO:0042803 | Protein homodimerization activity | 11 of 709 | 0.88 | 0.86 | 8.73e - 05 |
| GO:0042826 | Histone deacetylase binding | 5 of 125 | 1.29 | 0.82 | 0.0026 |

**Cellular Component (Gene Ontology)**

| **GO - term** | **description** | **count in network** | **strength** | **signal** | **false discovery rate** |
| --- | --- | --- | --- | --- | --- |
| GO:1990647 | C/EBP complex | 2 of 2 | 2.69 | 0.73 | 0.0114 |
| GO:0036488 | CHOP - C/EBP complex | 2 of 3 | 2.52 | 0.7 | 0.0136 |
| GO:0097418 | Neurofibrillary tangle | 2 of 5 | 2.29 | 0.63 | 0.0214 |
| GO:0036454 | Growth factor complex | 2 of 7 | 2.15 | 0.56 | 0.0325 |
| GO:0036477 | Somatodendritic compartment | 9 of 855 | 0.71 | 0.46 | 0.0136 |

**KEGG**

| **pathway** | **description** | **count in network** | **strength** | **signal** | **false discovery rate** |
| --- | --- | --- | --- | --- | --- |
| hsa04068 | FoxO signaling pathway | 7 of 126 | 1.44 | 1.69 | 1.40e - 06 |
| hsa04630 | JAK - STAT signaling pathway | 7 of 158 | 1.34 | 1.51 | 3.58e - 06 |
| hsa01524 | Platinum drug resistance | 5 of 70 | 1.55 | 1.45 | 2.76e - 05 |
| hsa05200 | Pathways in cancer | 12 of 515 | 1.06 | 1.39 | 1.10e - 07 |
| hsa04211 | Longevity regulating pathway | 5 of 87 | 1.45 | 1.31 | 6.38e - 05 |

**Supplementary Table 1:** GO and KEGG of Charts of GO and KEGG analysis of PPI - related interacting proteins.
