# Supplementary material for: Identification of senescence-related genes in diagnosing idiopathic pulmonary fibrosis via integrating bioinformatics analysis and machine learning
Source: PLoS One. 2026 Aug 7;21(8):e0353694. doi: 10.1371/journal.pone.0353694 (PMC13450802; doi:10.1371/journal.pone.0353694)
Supplement: Supplementary file 3 [file pone.0353694.s005.docx]

| **Protein** | **PDB/Uniprot ID** | **Small Molecule** | **CID** | **Score** | **Center (x, y, z)** | **Docking size (x, y, z)** |
| --- | --- | --- | --- | --- | --- | --- |
| CHEK2 | 2CN5 | Fostamatinib | 11671467 | -7.1 | 81, -45, 17 | 26, 26, 26 |

**Supplementary Table 2: Molecular docking results and scoring.**
